# CONTEXTUAL FACTORS RELATED TO CAREGIVER IDENTITY DISCREPANCY IN EMERGING ADULT CAREGIVERS

**DOI:** 10.1093/geroni/igac059.2112

**Published:** 2022-12-20

**Authors:** Anastasia Canell, Grace I L Caskie

**Affiliations:** Lehigh University, Bethlehem, Pennsylvania, United States; Lehigh University, Bethlehem, Pennsylvania, United States

## Abstract

Approximately 12-18% of unpaid family caregivers to older adults in the U.S. are 18-25 years old, yet minimal research focuses on this subgroup of caregivers (Levine, 2005). Because caregiver identity theory postulates that the extent to which informal caregivers integrate their caregiving roles and duties into their identity may relate to caregiver distress (Montgomery & Kosloski, 2012), contextual factors related to higher reported caregiver identity discrepancy should be assessed. In a sample of 135 emerging adult informal caregivers, the current study compared caregiver identity discrepancy across two factors: level of caregiving responsibility and caregiver gender. Caregiving responsibility had four groups: (1) primary with minimal help (n=54), primary but receiving some help (n=51), secondary (n=18), or shared (n=12). Caregiver identity discrepancy was measured using the five subscales of the Family Caregiver Identity Scale (Eifert et al., 2019). Caregiver identity discrepancy differed significantly by caregiving responsibility (p<.001) and gender (p=.046). Emerging adult caregivers identifying as women reported statistically more caregiver identity discrepancy in family obligation (p=.003) and master identity (p=.036) domains than men. Caregiving responsibility showed statistically significant differences in role engulfment (p<.001), loss of shared identity (p<.001), family obligation (p=.013), and master identity (p=.010) domains of identity discrepancy. Emerging adult caregivers identifying as the primary caregiver with minimal help from others reported significantly more identity discrepancy than all other groups across subscales. These results demonstrate that young caregivers who have little support in task responsibilities and identify as a woman may be at particular risk for experiencing caregiver identity discrepancy.

